# Adalimumab - an effective and promising treatment for patients with fistulizing Crohn's disease: a case series

**DOI:** 10.1186/1752-1947-5-109

**Published:** 2011-03-19

**Authors:** George Kouklakis, Eleni I Efremidou, Peter Zezos, Nikolaos Liratzopoulos, Vassilios D Souftas, Anthia Gatopoulou, Konstantinos Simopoulos, Konstantinos J Manolas

**Affiliations:** 1 Endoscopy Unit, 1st Surgical Department, Medical School, Democritus University of Thrace, University Hospital of Alexandroupolis, University Campus, Dragana 681 00 Alexandroupolis, Greece; 2 1st Surgical Department, Medical School, Democritus University of Thrace, University Hospital of Alexandroupolis, University Campus, Dragana 681 00, Alexandroupolis, Greece; 3 Department of Radiology, Medical School, Democritus University of Thrace, University Hospital of Alexandroupolis, University Campus, Dragana 681 00, Alexandroupolis, Greece; 4 2nd Surgical Department, Medical School, Democritus University of Thrace, University Hospital of Alexandroupolis, University Campus, Dragana 681 00, Alexandroupolis, Greece

## Abstract

**Introduction:**

Crohn's disease is a chronic inflammatory bowel disease of unknown etiology which may affect any part of the bowel. Fistulas are a common and often serious complication of Crohn's disease. The treatment for fistulizing Crohn's disease can be medical, surgical or a combination of the two. Recently, adalimumab, a fully human anti-tumor necrosis factor monoclonal antibody, has been suggested as a safe and effective treatment for the induction and maintenance of remission in adult patients with moderate to severe Crohn's disease, who are refractory to conventional therapy or intolerant to infliximab. However, large studies focusing on evaluating the efficacy of adalimumab in fistulizing Crohn's disease have not yet been published.

**Case presentation:**

We report the cases of three patients, of European Caucasian ethnicity and Greek nationality, with active luminal and fistulizing Crohn's disease. All of the cases were treated successfully with adalimumab. Patient 1 (a 44-year-old man) and patient 2 (an 18-year-old woman) developed early post-surgical enterocutaneous fistulas, while patient 3 (a 20-year-old woman) had peri-anal fistulizing Crohn's disease. Adalimumab treatment (160 mg subcutaneously at week zero, 80 mg at week two, and 40 mg every other week) was used for three different indications: (1) after the failure of other conservative medical treatments for Crohn's disease (patient 1); (2) as a monotherapy in treating a naive patient (patient 2); (3) after an intolerance to infliximab (patient 3). A remission of the active luminal and fistulizing disease was achieved soon after the initiation of adalimumab and sustained thereafter with maintenance doses. No further surgical intervention was required and no adverse effects were observed in any of the cases.

**Conclusions:**

Fistulizing Crohn's disease remains a challenge in clinical practice. Adalimumab seems to be an effective, well-tolerated and safe treatment option for the induction and maintenance of remission in patients with moderate to severe peri-anal fistulizing Crohn's disease. Furthermore, adalimumab seems to be a promising treatment option for patients with moderate to severe fistulizing Crohn's disease with enterocutaneous fistulas. However, this clinical observation needs to be investigated in further clinical trials.

## Introduction

Crohn's disease (CD) is a granulomatous, segmental, transmural inflammation of unknown etiology, affecting the bowel and predisposing the formation of strictures, perforation, abscesses and fistulas. Fistulas occur in 30 to 50 percent of CD patients during the course of disease. Peri-anal fistulas are the most common type (50 percent), followed by internal enteroenteric fistulas (25 percent) [1].

The treatment for fistulizing Crohn's disease (FCD) can be medical, surgical or a combination of the two. Various medical therapies, including antibiotics, immunomodulators (azathioprine, 6-mercaptopurine, cyclosporine) and total parenteral nutrition (TPN), have been used to treat FCD and have been effective to some degree. It is well-known that inflammation in CD is associated with high levels of tissue tumor necrosis factor-a (TNF-a) expression, and therapies directed against this cytokine have recently become the focus of interest. Infliximab, a chimeric (75 percent human and 25 percent murine) immunoglobulin G_1_(IgG_1_) monoclonal antibody against TNF-a, is the prototype anti-TNF-a agent shown to be efficacious in both the induction and maintenance of fistula closure in approximately two-thirds of patients and has now become the cornerstone of medical therapy for FCD [2]. However, some patients with FCD are refractory or intolerant to this agent. Recently, adalimumab (D2E7/Humira^®^, Abbott Laboratories), a fully humanized, subcutaneously-delivered immunoglobulin G_1_(IgG_1_) monoclonal antibody, which binds with high affinity and specificity to TNF but not to lymphotoxin, has been proven to be a safe and effective treatment for the induction and maintenance of remission in adult patients with moderate to severe CD (luminal and/or fistulizing), who are refractory to conventional therapy or intolerant to infliximab [3-5].

In the ACCENT II trial, Sands *et al. *evaluated and proved the efficacy of infliximab as a maintenance therapy for CD patients with fistulas [2]. Although previous studies which evaluated adalimumab included patients with FCD [3-5], there is one study published by Hinojosa *et al. *that clearly demonstrates the efficacy of adalimumab treatment in patients with luminal and/or FCD [6]. There is also published data about the efficacy of anti-TNF therapy, including adalimumab, in the sub-group of patients with peri-anal FCD [7,8].

In this case series, we describe our experience with adalimumab in the treatment of three adult patients with FCD that resulted in rapid fistula closure and sustained luminal disease remission.

## Case presentation

We report the cases of three patients, of European Caucasian ethnicity and Greek nationality, with active luminal and FCD.

### Patient 1

A 44-year-old man with a small bowel obstruction (SBO) underwent a laparotomy which revealed a stenosing, edematous small bowel segment near a former anastomosis. A total of 50 cm of his small bowel was resected. He had previously undergone an extensive surgical resection of the ileum for SBO. Both histologic examinations were suggestive of ischemic enteritis.

One week later, post-operative enterocutaneous fistulas (ECF) developed next to the drainage catheters originating from the anastomosis site. We administered TPN and antibiotics (ciprofloxacin 1000 mg/day and metronidazole 1500 mg/day for two weeks). One month later, the fistulas were still active, with draining of fecal-mucopurulent discharge as a byproduct of the fistulas daily upon oral feeding. An endoscopic examination revealed linear, deep ulcers with edematous margins in his ascending colon, cecum, ileocecal valve and terminal ileum. A histologic examination revealed a mild derangement of the enteric crypts architecture, moderate inflammatory focal cryptitis, neutrophilic and eosinophilic infiltration, and glandular abscesses without mucus. The findings were consistent with the diagnosis of CD and treatment with adalimumab subcutaneous injections was initiated (160 mg at week zero, 80 mg at week two, and 40 mg every other week). Drainage from all fistulas was stopped one week after the first dose, while a complete closure of the fistulas was achieved at week six and complete remission of the mucosal lesions was observed in an endoscopy after 14 weeks of treatment. He is currently in remission. This is maintained with adalimumab monotherapy at 40 mg subcutaneously every other week, without any adverse effects.

### Patient 2

An 18-year-old woman presenting with fever (38.2°C) and localized right lower quadrant pain underwent surgery for acute appendicitis. During a laparotomy, severe inflammation of her cecum and terminal ileum was observed, but only an appendicectomy was performed. Ten days later, a post-operative fistula developed at the surgical wound with a fecal-purulent discharge. A colonoscopy revealed linear ulcers in her ileum and ascending colon. A histologic examination revealed focal ulceration of the surface epithelium, derangement of the architecture of enteric crypts, severe cryptitis, and a moderate inflammatory infiltration of the lamina propria with neutrophil leucocytes, lymphocytes, plasma cells and eosinophils and epithelioid granulomas. She was treated with adalimumab monotherapy at a dosage of 160 mg at week zero, 80 mg at week two, and 40 mg every other week. Two weeks after the third injection, (in the sixth week of treatment) the fistula was completely healed, while an endoscopy showed healing of the gastrointestinal ulcers and luminal disease remission. She is currently undergoing maintenance treatment with adalimumab at 40 mg every other week.

### Patient 3

A 20-year-old woman was referred to our endoscopy unit with five peri-anal fistulizing ducts which had developed during a two-year course of fever and bloody diarrhea. A colonoscopy revealed linear ulcers in her terminal ileum, cecum and ascending colon. A histologic examination of the biopsy samples showed mucosal surface erosions and dense inflammatory cellular infiltrations of lymphocytes, plasma cells, eosinophils and neutrophil leucocytes in the lamina propria extending to the muscularis mucosa and crypt abscesses. A diagnosis of CD was confirmed with a Crohn's Disease Activity Index (CDAI) score of 320 and a Peri-anal Disease Activity Index (PDAI) score of 14. An examination under anesthesia revealed complex peri-anal disease and non-cutting drainage setons were inserted in the fistula tracks. Consequently, she was treated with mesalamine (3.2 g/d), oral prednisolone (1 mg/kg) with a tapering schedule, metronidazole (1500 mg/d for 10 days) and ciprofloxacin (1000 mg/d for one month). Three months later, there had been no significant improvement and she continued to require high doses of prednisolone (20 mg/d). A steroid-sparing treatment with azathioprine (100 mg/day) and later with methotrexate (15 mg/wk) failed because of serious drug-induced adverse effects; she developed pancreatitis after six weeks of azathioprine and hepatitis after three weeks of methotrexate. Finally, infliximab (5 mg/kg at weeks zero, two, and six, and then every eight weeks) was administered and resulted in the closure of two of five draining fistula ducts (PDAI score: 7). Unfortunately, during the sixth injection (in the 30th week of treatment), she developed severe acute laryngismus with hoarseness, dyspnea, cyanosis and tachycardia. The infliximab treatment was discontinued. Four weeks later, an endoscopic evaluation showed a recurrence of active luminal CD, while the peri-anal disease had not deteriorated (PDAI score: 7). Treatment with per-oral prednisolone (1 mg/kg) was administered in combination with adalimumab subcutaneous injections at a dosage of 160 mg at week zero, 80 mg at week two and 40 mg every other week. This combination of treatment resulted in a complete closure of the fistulas after seven weeks (PDAI score: 0) and complete remission of luminal CD (CDAI score: 132). Subsequently, the corticosteroids were discontinued while adalimumab was continued as a maintenance treatment at a dose of 40 mg every other week. She has experienced complete fistula closure for the last 18 months without any adverse effects.

## Discussion

Our case report describes the cases of three patients with both active luminal and FCD who were treated successfully with adalimumab (Table 1). Patient 3, had severe peri-anal FCD, while patients 1 and 2 developed post-operative ECF. In the latter two cases, the development of fistulas was the reason for further endoscopic investigation and the final diagnosis of CD.

**Table 1:** Demographic and clinical details of patients treated with adalimumab

Number	Case	Luminal + FCD	Fistula type	Previous failed and/or intolerant therapies	Current treatment	Adverse effects	Treatment outcome
1	44, M ileo-colonic CD	Yes	Enterocutaneous, post-operative	Parenteral nutrition Antibiotics - ciprofloxacine and metronidazole	Adalimumab monotherapy [160-80-40]	no	Luminal disease remission Fistula healing
3	20, F ileo-colonic CD peri-anal disease	Yes	Peri-anal	Corticosteroid resistant Antibiotics - ciprofloxacine and metronidazole Azathioprine (fever and pancreatitis) Methotrexate (hepatitis) Reaction to infliximab (laryngismus)	Corticosteroids (tapered off) plus Adalimumab [160-80-40] Seton drainage	no	Luminal disease remission Fistula healing
2	18, F ileo-colonic CD	Yes	Enterocutaneous, post-operative	No	Adalimumab monotherapy [160-80-40]	no	Luminal disease remission Fistula healing

CD: Crohn's disease; M: male; F: female

In patients 1 and 2, adalimumab treatment was used for three different indications: (1) the failure of other conservative medical treatments for CD (patient 1); as monotherapy in treating a naive patient (patient 2); following an intolerance to infliximab (patient 3). Treatment with adalimumab was proven to be efficacious and safe in all of the three cases.

Remission of active luminal and fistulizing disease was achieved soon after the initiation of adalimumab and sustained thereafter with maintenance doses of adalimumab. No further surgical intervention was necessary and no adverse effects were observed in any of the cases.

The treatment of fistulas, which complicate CD in up to 40 percent of cases, has greatly evolved in the last 15 years, mainly because of improvements in medical therapy including immunomodulators (azathioprine, methotrexate) and biologics (infliximab, adalimumab) [7,8]. The advent of immunomodulators and, later, anti-TNF-a agents has positioned conservative medical therapy as the first-line treatment for CD fistulas, with surgery reserved for refractory or complicated cases. Several published studies show that TNF-a antagonists (anti-TNF-a) are effective in inducing and maintaining disease remission in patients with CD. Infliximab has been shown to be effective for the treatment of FCD in adult patients with peri-anal fistulas. Infliximab has even been shown to sustain a response in patients who had an initial clinical response with fistulas closure to induction therapy [7,8].

The ACCENT II trial assessed the efficacy of infliximab maintenance therapy in adult patients with CD who had at least one draining abdominal or peri-anal fistula over a period of at least three months [2]. All patients received an induction dose of 5 mg/kg in weeks zero, two, and six and afterwards they were randomized to either a placebo or infliximab maintenance therapy (5 mg/kg every eight weeks) for a total of 54 weeks. The data indicated that a maintenance treatment with infliximab was superior to the placebo in patients who responded to infliximab induction therapy at weeks zero, two and six. At week 54, 19 percent of patients receiving the placebo had complete fistula closure compared with 36 percent of patients in the infliximab maintenance group (*p *= 0.009). More recently, adalimumab, another TNF-a antagonist, has been approved in the US and EU for the treatment of CD. Clinical trials have demonstrated that adalimumab is both effective for the induction and maintenance of remission in patients with moderate to severe CD, and safe and efficacious in regaining a medical response in patients intolerant or non-responsive to infliximab [3-5,7,8].

In the CHARM trial, the primary objective was to assess the benefit of two adalimumab dosing regimens - 40 mg every other week and weekly - in maintaining clinical remission at 26 and 56 weeks in patients with moderate to severe CD. Overall efficacy in fistula closure was also assessed, with significant effects of adalimumab on fistula closure observed at 26 and 56 weeks. Complete fistula closure was observed more frequently among patients treated with adalimumab than those who were receiving a placebo: 30 percent and 13 percent, respectively, at week 26 (*p *= 0.043) and 33 percent and 13 percent, respectively, at week 56 (*p *= 0.016). At week 56, fistula closure was maintained in 100 percent of patients who had complete fistula closure at week 26 [4,8].

Hinojosa *et al. *also evaluated adalimumab in a sub-set of 22 patients with FCD who lost response to or could not tolerate infliximab. After adalimumab induction therapy with 160 mg at week zero and 80 mg at week two, 23 percent of patients experienced fistula remission and 41 percent experienced fistula improvement at week four [6]. In addition, adalimumab has been reported as an effective and safe treatment in a pediatric CD patient with severe refractory luminal and fistulizing disease [9].

Recently-reported data from an extension of the CHARM trial includes a description and analysis of the demographics, disease characteristics and outcome in the sub-group of patients with fistulas treated with adalimumab, along with an evaluation of the two-year maintenance of fistula closure during treatment with adalimumab (ADHERE trial) [7]. This analysis has shown that adalimumab therapy was associated with progressive increases in fistula closure over time, with statistically significant differences between the placebo and adalimumab groups (p < 0,05) which were first observed at 16 weeks (15 percent in the placebo group versus 36 percent in the adalimumab group) [7]. For all randomly-assigned patients, there was a significant decrease in the mean number of draining fistulas per day among patients treated with adalimumab by comparison with patients treated with a placebo during the double-blind treatment period (0,88 for the adalimumab group versus 1,34 for the placebo group; p = 0,002). Approximately 60 percent of the patients with FCD who were treated with adalimumab had healed fistulas after two years of therapy, while 90 percent of patients with healed fistulas at the end of the CHARM trial maintained closure following one additional year of treatment in the ADHERE trial [7]. The effect of adalimumab on the number of draining fistulas in each sub-group (based on immunosuppressants, antibiotics or previous TNF antagonists experience) was similar to that observed for the placebo and adalimumab groups [7]. This recent data shows that adalimumab therapy for FCD resulted in a significant and complete healing of draining fistulas, with a safety profile consistent with previous studies on patients with active CD [8].

Adalimumab is also a well-tolerated treatment for CD in patients who are infliximab naive and for those who do not respond to or are intolerant of infliximab [4,5,7]. In patients treated with adalimumab, reactions at the injection site are the most common adverse effects, while large series show low rates of serious adverse effects similar to a placebo [4,5,7,8]. Adalimumab also offers the benefits of decreased immunogenicity and the ease of subcutaneous administration. Data from the CLASSIC II trial demonstrated that the percentage of patients developing antibodies to adalimumab was low (2.6 percent) [5].

In the case of patient 3, adalimumab therapy resulted in complete disease remission and the closure of peri-anal fistulas. This fistula healing was maintained for 18 months without adverse effects.

Although adalimumab has been evaluated for its efficacy in the healing of peri-anal fistulas in active CD, there is, as yet, no published data on the use of adalimumab in patients with ECF. In general, patients with inflammatory bowel disease represent a high-risk group for the formation of ECF, as a result of both the process of their disease and the need for multiple surgeries [10-12].

ECF are one of the most complex and challenging complications encountered in surgical practice. The majority of them (75 to 85 percent) are post-operative in origin, while spontaneous fistulas account for 15 to 25 percent of all ECF [12-14]. Due to several advances in medical treatment, the rates of spontaneous fistula closure have improved significantly, while mortality remains high, ranging between 5 and 25 percent [15].

The current management of ECF includes controlling sepsis, optimization of the nutritional state, wound care, assessment of the fistula anatomy, and the timing of surgery and surgical strategy (the SOWATS guideline), with priority given to sepsis treatment and restoration of the nutritional state [12-15]. Independent variables examined in reported studies, which are essential in promoting the spontaneous closure of ECF, include control of malnutrition, hydroelectrolytic imbalance, serum albumin and elimination of sepsis [14]. In addition, it is generally recommended that patients do not undergo restorative surgery in the three to six month period after the development of ECF [12-15]. Earlier studies also report that the spontaneous closure of ECF is less common in fistulas caused by malignancy or CD and is predominantly seen in colonic ECF, in low-output ECF and in patients with a closed abdomen [13,14]. Moreover, the probability and timing of a spontaneous closure of post-operative ECF is related to the location of the fistula, the site of origin, the output during 24 hours, the type of post-operative ECF and the fistulous tract. Overall, small bowel post-operative ECF have a lower chance of spontaneous closure (~30 percent) compared to colonic fistulas (~85 percent), while jejunal fistulas have a higher rate (~40 percent) compared to ileal fistulas (~25 percent). Multiple post-operative ECF, and those with a complex fistulous tract and high output (>500 ml/24 hours), have a low rate of spontaneous closure (3 and 2,5 times higher versus single and low-output post-operative ECF, respectively). Due to these multiple variables, the rates of spontaneous closure of post-operative ECF vary (17 to 71 percent) (Table 2) [12-14].

**Table 2:** Treatment management of ECF and outcomes

Treatment	ECF outcome
SOWATS treatment guideline (medical + surgical treatment)	Mortality: 10-30% Fistula closure: ~87,4%

Medical treatment **Alimentary tract rest** **Restoration of circulating volume** **Restoration of hydroelectrolytic balance** **Nutritional support **(optimal TPN) (fistula closure rate: 89-92,3%) **Controlling sepsis** **Wound care**	Mortality: 5-25% Spontaneous closure: 17-71%

Restorative surgery Minimal period: >6 weeks	Mortality: 9,6%-10,8% (sepsis-related death: ~15,1%) Surgical closure: 52-90,7% Persisting fistula: 3-50%
Definitive surgery Minimal period: >6 months (median period: 8 months)	

Small bowel ECF	Mortality: ~35% Spontaneous closure: ~30%

Colonic ECF	Mortality: ~22% Spontaneous closure: ~85%

References: [12-15]

In our case report, we describe the cases of two patients who developed post-operative ECF refractory to classical management with TPN, antibiotics, octreotide and wound care. The sites of origin were the small bowel (patient 1) and the cecum (patient 2) and there were simple fistulous tracts in both cases. Further investigation led to a diagnosis of active CD and treatment with a TNF-a antagonist was initiated. Both patients received adalimumab. We achieved a remission of the disease and a complete spontaneous closure of the post-operative ECF in both cases.

## Conclusions

The results from a meta-analysis by Peyrin-Biroulet *et al. *demonstrate that anti-TNF therapy is safe and effective for both luminal and FCD, in patients who are refractory to standard medical therapy. Due to the small sample size of these drugs, it was not possible to perform a sub-group analysis or a direct comparison between anti-TNF agents, however, in an overall analysis, anti-TNF therapy was more effective than a placebo for fistula healing in FCD. The above, more detailed, analysis elevated the results from the ACCENT II study on the safety and efficacy of infliximab in inducing and maintaining fistula closure [8]. The CHARM trial, and more recently the study by Colombel *et al. *regarding the ADHERE study, showed that adalimumab therapy results in the significant and complete healing of draining fistulas in active FCD. Furthermore, the long-term healing of draining fistulas was maintained over two years from the baseline of the CHARM study, while in the fistula cohort there were no differences in the rate of adverse effects in patients who received adalimumab compared to those who received a placebo [7].

The presence of active inflammation as a result of CD, following on from or without surgery, predisposes to fistulation. An early diagnosis of ECF, the resuscitation of the patient, the provision of nutritional support and the control of sepsis have decreased morbidity and mortality rates and often result in a spontaneous closure of fistulas. Definitive surgical management should be performed only after the restitution of normal physiology and nutritional improvement; usually after at least six months, but often over a longer period [12-15].

Our goal in this case report was to audit the results of adalimumab therapy in patients with moderate to severe FCD. Although our case series cannot safely propose a treatment approach, our secondary aim was to report on both the spontaneous closure of post-operative ECF with adalimumab therapy in patients with severe active CD, and adalimumab as a monotherapy treatment for the long-term maintenance of both a remission of CD and the healing of fistulas. Our main message is that the combination of adalimumab therapy and a standardized management protocol for ECF can result in a good patient outcome in cases of FCD. Nevertheless, larger studies focusing on the evaluation of adalimumab treatment in patients with FCD, especially patients who develop spontaneous or post-operative ECF, are needed to expand on the present experience of treatment in this sub-population of CD.

## Consent

Written informed consent was obtained from the patients for publication of this case report and any accompanying images. A copy of the written consent is available for review by the Editor-in-Chief of this journal.

## Competing interests

The authors declare that they have no competing interests.

## Authors' contributions

GK conceived of the study and participated in its design and coordination. EIE participated in the design of the study, the acquisition and interpretation of the data and the drafting of the manuscript. PZ participated in the sequence alignment and the drafting of the manuscript. NL participated in the design of the study and the coordination and helped to revise the manuscript. VDS participated in the coordination and acquisition of data. AG participated in the sequence alignment and interpretation of data. KS revised the manuscript for the intellectual content and gave final approval of the version to be published. KJM participated in the conception of the study, revised the manuscript for the intellectual content and gave final approval of the version to be published. All authors read and approved the final manuscript.
